# Etymologia: *Acanthamoeba*

**DOI:** 10.3201/eid2608.ET2608

**Published:** 2020-08

**Authors:** Nitika Pradhan

**Affiliations:** Kalinga Institute of Industrial Technology, Bhubaneswar, India

**Keywords:** *Acanthamoeba*, amoeba, protozoa, protist, spine-like structures, granulomatous encephalitis, keratitis, acanthamebiasis

## *Acanthamoeba* [ǝˌ́́́́kæn.Өǝʹmi.bǝ]

From the Greek akantha (spike/thorn), which was added before amoeba (change) to describe this organism as having a spine-like structure (acanthopodia). This organism is now well-known as *Acanthamoeba*, an amphizoic, opportunistic, and nonopportunistic protozoan protist widely distributed in the environment.

In 1930, it was reported by Castellani in yeast (*Cryptococcus pararoseus*) culture, and was later (1931) classified as the genus *Acanthamoeba* by Volkonsky. It was later found to be the etiologic agent of *Acanthamoeba* granulomatous encephalitis and keratitis in humans. This organism can also cause cutaneous acanthamebiasis in debilitated and immunocompromised patients (Figure).

**Figure:**
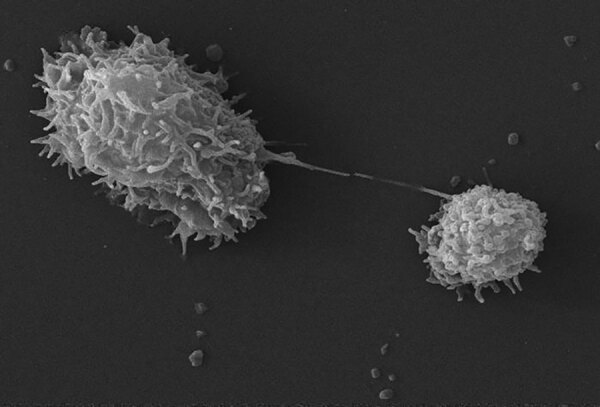
This scanning electron microscopic image shows an *Acanthamoeba polyphaga* protozoa about to complete the process of cell division known as mitosis, thereby becoming 2 distinct organisms. Note the numerous pseudopodia projecting from the surfaces of these organisms. These pseudopodia enable the amoebae to move about and grasp objects in their environment. Source: Centers for Disease Control and Prevention/Catherine Armbruster, Margaret William; photograph, Janice Haney Carr, 2009.
